# Dissection of the practical soybean breeding pipeline by developing ZDX1, a high-throughput functional array

**DOI:** 10.1007/s00122-022-04043-w

**Published:** 2022-02-21

**Authors:** Rujian Sun, Bincheng Sun, Yu Tian, Shanshan Su, Yong Zhang, Wanhai Zhang, Jingshun Wang, Ping Yu, Bingfu Guo, Huihui Li, Yanfei Li, Huawei Gao, Yongzhe Gu, Lili Yu, Yansong Ma, Erhu Su, Qiang Li, Xingguo Hu, Qi Zhang, Rongqi Guo, Shen Chai, Lei Feng, Jun Wang, Huilong Hong, Jiangyuan Xu, Xindong Yao, Jing Wen, Jiqiang Liu, Yinghui Li, Lijuan Qiu

**Affiliations:** 1 College of Agriculture, Northeast Agricultural University, Harbin, 150030 People’s Republic of China; 2 National Key Facility for Crop Gene Resources and Genetic Improvement, Institute of Crop Sciences, Chinese Academy of Agricultural Sciences, No.12 Zhongguancun South Street, Haidian District, Beijing, 100081 People’s Republic of China; 3 Hulunbuir Institute of Agriculture and Animal Husbandry, Hulunbuir, 021000 People’s Republic of China; 4 Beijing Compass Biotechnology Co, Ltd, Beijing, 102200 People’s Republic of China; 5 Keshan Branch of Heilongjiang Academy of Agricultural Sciences, Qiqihar, 161600 People’s Republic of China; 6 Inner Mongolia Academy of Agricultural and Animal Husbandry Sciences, Hohhot, 010000 People’s Republic of China; 7 Department of Crop Sciences, University of Natural Resources and Life Sciences Vienna (BOKU), 3430 Tulln, Austria

## Abstract

**Key message:**

We developed the ZDX1 high-throughput functional soybean array for high accuracy evaluation and selection of both parents and progeny, which can greatly accelerate soybean breeding.

**Abstract:**

Microarray technology facilitates rapid, accurate, and economical genotyping. Here, using resequencing data from 2214 representative soybean accessions, we developed the high-throughput functional array ZDX1, containing 158,959 SNPs, covering 90.92% of soybean genes and sites related to important traits. By application of the array, a total of 817 accessions were genotyped, including three subpopulations of candidate parental lines, parental lines and their progeny from practical breeding. The fixed SNPs were identified in progeny, indicating artificial selection during the breeding process. By identifying functional sites of target traits, novel soybean cyst nematode-resistant progeny and maturity-related novel sources were identified by allele combinations, demonstrating that functional sites provide an efficient method for the rapid screening of desirable traits or gene sources. Notably, we found that the breeding index (BI) was a good indicator for progeny selection. Superior progeny were derived from the combination of distantly related parents, with at least one parent having a higher BI. Furthermore, new combinations based on good performance were proposed for further breeding after excluding redundant and closely related parents. Genomic best linear unbiased prediction (GBLUP) analysis was the best analysis method and achieved the highest accuracy in predicting four traits when comparing SNPs in genic regions rather than whole genomic or intergenic SNPs. The prediction accuracy was improved by 32.1% by using progeny to expand the training population. Collectively, a versatile assay demonstrated that the functional ZDX1 array provided efficient information for the design and optimization of a breeding pipeline for accelerated soybean breeding.

**Supplementary Information:**

The online version contains supplementary material available at 10.1007/s00122-022-04043-w.

## Introduction

The goal of crop breeding is to develop plant varieties with ideal traits, such as higher yield, improved quality, and enhanced environmental adaptability.﻿ The yield of soybean [*Glycine max* (L). Merr.] increase per unit area has not been improved significantly during the past few decades (Liu et al. 2020) due to the limitation of traditional phenotyping methods to develop new varieties (Barabaschi et al. 2016). Innovative genotyping platforms can accelerate the process of identification, evaluation, and use of elite germplasm resources (Bailey-Serres et al. 2019; Viquez-Zamora et al. 2013; Yu et al. 2014).

The publication of the soybean genome has facilitated the discovery of single nucleotide polymorphisms (SNPs) (Schmutz et al. 2010), and SNP arrays have become a key technology in soybean genetics research. Despite low SNP density, previously developed soybean arrays have been used for research, including diversity analysis, genetic mapping, and association analysis (Hyten et al. 2008; Song et al. 2020; Wang et al. 2018b). More recently, the 50 K soybean array, which has higher density, was used to genotype 96 elite, landrace, and wild accessions, and to identify candidate genomic regions shaped by domestication or recent selection (Song et al. 2013). Similarly, this array was used to correlate protein- and oil-related loci via genome-wide association study (GWAS) analysis of 298 strains (Hwang et al. 2014). When 180 K (Lee et al. 2015) and 355 K (Wang et al. 2016) arrays were developed, natural hybrids between cultivated and wild soybean, as well as a candidate interval affecting grain weight, were identified. These arrays laid the foundation for the application of SNP arrays in genetic research and molecular breeding.

One of the key challenges facing plant breeders is the selection of suitable parents to generate sufficiently rich genetic variation to allow a maximal selection response during the breeding cycle in self-pollinating crops (Ji et al. 2018). To meet this challenge, new and more effective breeding strategies that combine phenotypic data with high-throughput genotyping should be developed to better identify prospective germplasm and to evaluate progeny (Varshney et al. 2014). Soybeans of different types (Pandey et al. 2017) and from different sources (Marrano et al. 2019) can be distinguished using microarrays to provide a basis for determining the most suitable parents. Molecular markers associated with agronomically valuable traits that are not easily scored can also help in the early evaluation of parents and the identification of desirable progeny (Rasheed et al. 2017). With the development of microarrays, genomic selection based on a large number of markers can be more informative and robust in selecting for complex traits controlled by multiple genes, such as yield, seed quality, and disease resistance (Xu et al. 2020). However, there are relatively few reports describing how to integrate high-throughput sequencing into the main breeding process.

Currently, there is an urgent need to develop a functional SNP array that covers the entire soybean genome and also contains representative and important sites to facilitate genetic research and molecular breeding. Here, we screened representative SNPs from a wide range of soybean accessions and developed the “Zhongdouxin No.1” (ZDX1) functional array. Using a breeding population comprised of 817 accessions, including candidate parental line subpopulations, parental lines, and their derived progeny subpopulations, we demonstrate the use of this array in improving steps in breeding, including screening for new genetic resources, population diversity analysis, optimizing hybrid combinations, and progeny selection. The ZDX1 array described in this work, with associated breeding selection strategies, can accelerate all of the steps in the breeding process.

## Materials and methods

### SNP detection, filtering, and selection for array development

**Fig. 1:**
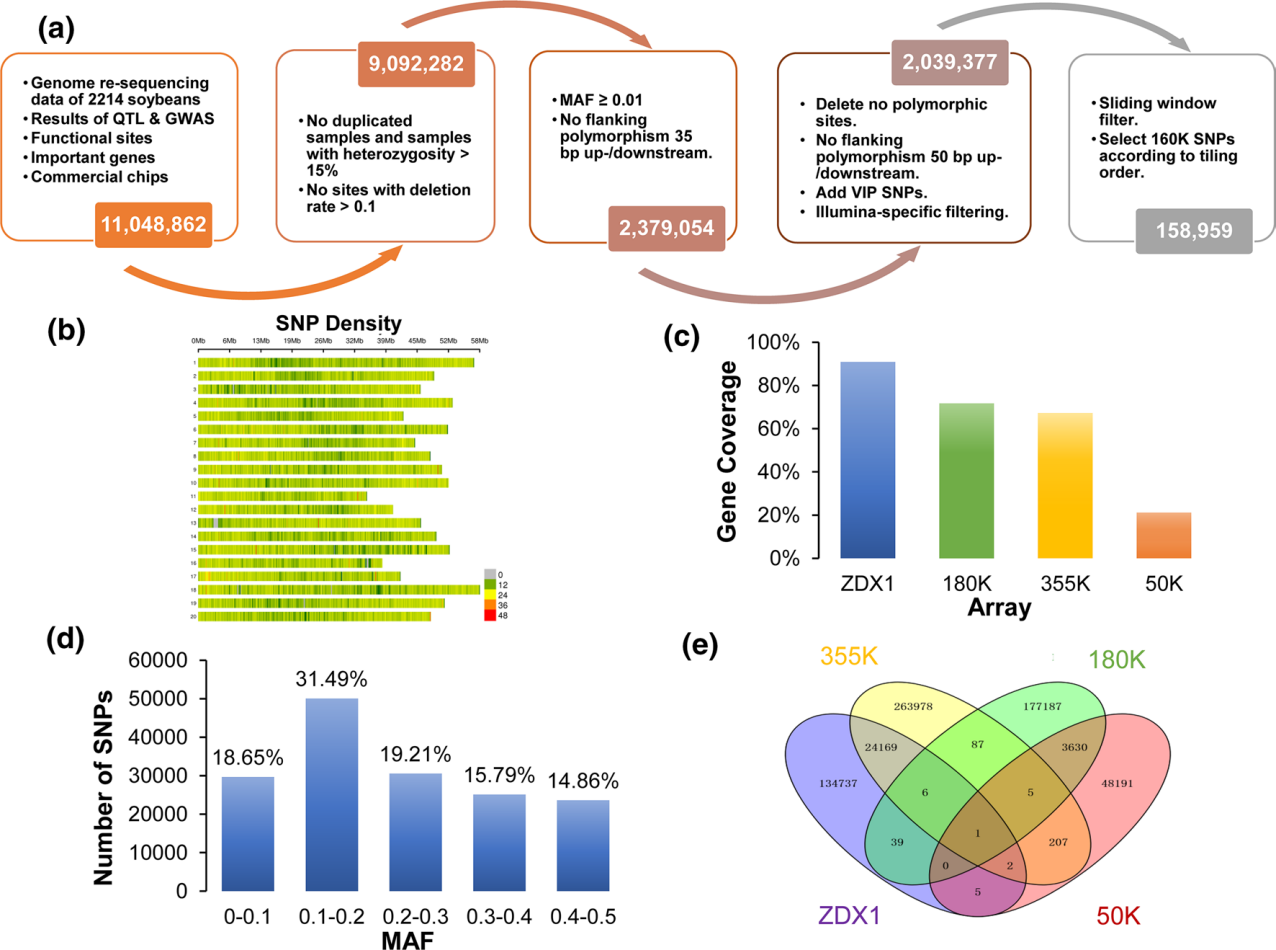
Summary information content of ZDX1 array. **a** Pipeline of single nucleotide polymorphism (SNP) identification and selection for the ZDX1 array. **b** The distribution of SNP loci on the soybean chromosomes. **c** The percentage of gene coverage in the ZDX1 array, the SoySNP50K array, the 180 K AXIOM^®^ array, and the NJAU 355 K SoySNP array. **d** The number of SNPs belonging to different minor allele frequency (MAF) classes based on 2214 soybean accessions. **e** Venn diagram showing the overlap of SNP positions between the ZDX1, SoySNP50K, 180 K AXIOM^®^, and NJAU 355 K SoySNP arrays

Using resequencing data from 2214 soybean accessions (including 862 improved cultivars (*Glycine max* (L.) Merr.), 1131 landraces, 218 annual wild soybean accessions (*Glycine soja* Sieb. & Zucc.), and three perennial wild soybean accessions (*Glycine* subgenus *Glycine*) as the basic information and based on the Illumina platform (Fig. S1), we obtained the VCF file by comparison with the reference genome Wm82.a2.v1 (Gmax_275_v2.0) and we also obtained 11,048,862 initial polymorphic SNP sites, including commercialized array sites, important gene sites, quantitative trait locus (QTL) and GWAS sites, and important trait functional sites. After the removal of sites with a deletion rate of > 0.1 and a degree of heterozygosity > 15%, 9,092,282 sites were retained. We then screened 2,379,054 sites according to the criteria of “retaining sites with MAF ≥ 0.01”. We deleted the sites with variants within 50 bp of the flanking regions, keeping the tiling order = 1 site, and 2,039,377 sites remained. Based on 2214 soybean accessions, we deleted the sites with errors, tested 41 sliding window gradients for site screening, and selected a 4800-bp window. The principle for site selection was “priority + Illumina score ≥ 0.4 + non-AT/GC selection site (if there is no non-AT/GC, then select the sites with higher priority),” among which the priority definition principles were: I. excellent QTL sites, GWAS sites, important genes, selective genes, genome-wide genes, and terminator/alternative splicing/nonsynonymous mutation sites; II. interspecies and intraspecific subgroup unique sites; III. selection interval (domestication) sites; IV. whole-genome coverage sites; and V. gap-filling sites. Finally, 158,959 SNP sites were obtained for ZDX1 (Fig. 1a).

## Plant materials and phenotypic data collection

The plants used in this study consisted of 817 accessions from the actual breeding population, including 77 parental lines, 169 candidate parental lines, and 571 elite progeny. Progeny were stable lines obtained by the pedigree method after crossing. Among them, there were 298 progeny for which both parents were included in parental lines and 273 progeny for which only single parents (male or female) were included in parental lines. Additionally, 283 of the 571 progeny were bred in 2015, while 288 progeny were bred in 2016.

The field experiment with the 817 accessions was performed with three replicates (designated as environments L1 and L2) in Zhalantun City, Inner Mongolia (47°40′ N, 122°36′ E) in 2017 and 2018, and one replicate in Keshan County, Heilongjiang Province in 2018 (48°33′ N, 126°8′ E) (designated as environment L3). The experiment used a randomized block design and one control line (Neidou4hao or Keshan1hao) was planted for every 20 experimental lines, with a row spacing of 0.65 m, plant spacing of 0.05 m, and a row length of 3 m. One row was planted for each material, and the area of each plot was 1.95 square meters. The sowing dates in the three environments of L1, L2, and L3 were May 12, May 9, and May 7 for each year, and the emergence dates of seedlings were May 25, May 21, and May 19 each year. A total of six quantitative traits were investigated: These included VE, defined as the date of emergence of the cotyledons. Beginning maturity (R7) was defined as the days from emergence to when one pod on the main stem had reached a mature pod color (Fehr et al. 1971). For each row, the R7 date was defined as when 50% of the plants meet the above condition. In the middle of each plot, 20 plants were continuously harvested when there was no shortage of seedlings. The seed yield in each plot (SY), 100-seed weight (SW), protein content, and oil content were also measured. One qualitative trait, leaf shape, was recorded as either a narrow or broad leaflet (Qiu et al. 2020).

## Genotypic data collection

A commercial kit (Tiangen Plant Genomic DNA Kit, DP305) was used to extract genomic DNA from young soybean leaves. We used the ZDX1 SNP array developed based on the Illumina^®^ platform as a typing tool (Zhao et al. 2018), used GenomeStudio software to obtain the SNP genotypes (GenomeStudio 2008), tested and adjusted the typing signal, which was > 3 (Fig. S2). The ZDX1 array contained 14 reported functional loci, including six for the growth period, namely, *e1-fs*, *e1-as* (Tsubokura et al. 2014; Xia et al. 2012), *e3-fs* (Tardivel et al. 2014; Xu et al. 2013), *e4-keshuang* (Langewisch et al. 2014; Tsubokura et al. 2013), *e4-oto* (Langewisch et al. 2014; Tsubokura et al. 2013), and *GmGPRR3b/Tof12* (Li et al. 2020); three sites in genes for cyst nematode resistance, namely *rhg1-a/GmSNAP18* (Cook et al. 2012; Shi et al. 2015), *Rhg4/GmSHMT08* (Liu et al. 2012; Shi et al. 2015), and *GmSNAP11* (Tian et al. 2019, 2018); leaf shape *Ln*/*ln* (Jeong et al. 2012); stem termination, *Dt1/Gmtfl1-ta* and *Dt1/Gmtfl1-ab* (Langewisch et al. 2014; Tian et al. 2010); seed coat color, *Gm850* (Wang et al. 2018a); and seed coat gloss, *Bloom1* (Zhang et al. 2018).

## Population genetic analysis

PLINK v2.1.1 (Purcell et al. 2007) was used to control the genotypes. We screened out 7099 sites with a genotyping success rate of < 90%, eight Insertion/Deletion (Indel) sites, 745 sites on scaffolds, and 82,085 sites with MAF < 0.05. A total of 69,022 valid SNPs remained. Linkage disequilibrium (LD) analysis was performed with Ldheatmap software, in which the maximum distance (kb) between two SNPs was set to 1000, and the correlation coefficient (*r*^2^) of alleles was calculated to measure the LD in each group level. The LD decay rate was defined as the chromosomal distance at which the average *r*^2^ dropped to half its maximum value. The kinship matrix was calculated using the VanRaden method in Gapit software to obtain the genetic relationships between lines in the population.

To remove the SNPs whose LD is greater than 0.5 to any other SNPs in the window we defined with parental lines and candidate parental lines, the following method was used: (a) a window of 50 SNPs was considered; (b) the LD between each pair of SNPs in the window was calculated; (c) one of a pair of SNPs was removed if the LD was greater than 0.5; (d) the window was shifted five SNPs forward and the procedure was repeated. This method was used to obtain a total of 8940 loci. PLINK v2.1.1 was used for principal component analysis (PCA), and R software was used to draw PCA diagrams.

## Best linear unbiased estimates and breeding index

The R asreml data package was used to calculate the best linear unbiased estimates (BLUE) from the phenotypic data for genomic selection (He et al. 2016) and to provide a breeding index (BI).

For the BI, the index is a linear combination of the predicted values of comprehensive traits, with each having a unique weight, as follows:

$$I_{j} = \mathop \sum \limits_{k = 1}^{5} w_{k} \hat{y}_{jk}^{*}$$

where *I*_*j*_ is the selection index score for individual *j*, *w*_*k*_ is the economic weight for the kth trait for *k* = 1,2,…,5, and $\hat{y}_{jk}^{*}$ is the standardized predicted value for trait k from the *j*th individual accession, which is calculated by standardizing the values for each trait by subtracting the mean value and dividing by the SD. We included five traits in the selection index corresponding to the following order, R7, SW, protein, oil, and SY (Cui et al. 2019; Zhao et al. 2015). The weight of the five traits is shown below:

$$w \, = \, \left[ { - 0.2, \, 0.1, \, 0.2, \, 0.1, \, 0.4} \right]$$

Among the 246 parents, the BI of the top third, middle third, and bottom third from high to low was designated as high parents, medium parents, and low parents, respectively, with 82 accessions in each group. In addition, the term “rate over best-parent” meant the proportion of progeny with better performance than that of the “best” parent.

## Heritability and genomic selection

PLINK v2.1.1 was used to control the genotype, which left 69,022 valid SNPs remaining. Pedigree-based best linear unbiased prediction (ABLUP) (Song et al. 2019), genomic best linear unbiased prediction (GBLUP) (Zhe et al. 2015), and combined best linear unbiased prediction (HBLUP) (Li et al. 2014; Lourenco et al. 2020; Song et al. 2017) were performed by BGLR (Pérez and de Los Campos 2014), asreml (Gilmour et al. 2015), and R software, respectively. We computed the broad-sense heritability using the following formula in QTL ICIMapping (Meng et al. 2015):

$$H^{2} = \frac{{\sigma_{G}^{2} }}{{\sigma_{G}^{2} + \frac{{\sigma_{{{\text{GE}}}}^{2} }}{e} + \frac{{\sigma_{\varepsilon }^{2} }}{{{\text{er}}}}}}$$

where $\sigma_{G}^{2}$ is the variance among soybean lines, $\sigma_{GE}^{2}$ is the genotype-by-environment interaction variance, $\sigma_{\varepsilon }^{2}$ is the residual variation, and e and r are the number of environments and replications within environments, respectively.

The Pearson correlation coefficient between the predicted and observed phenotype (rMP) was estimated, and the prediction accuracy (rGS) was calculated for the standardized rMP by the square root of the broad-sense heritability (Lehermeier et al. 2013). When comparing the prediction effects of gene regions, intergenic regions, and whole-genome markers, the following strategies were adopted for marker sampling. Among the 69,022 loci retained after filtering, the number of gene regions was 33,756 and the number of intergenic regions was 35,266. To eliminate the influence of the number of loci on the prediction accuracy, all 33,756 of the loci were reserved in the gene regions, 33,733 of the loci were uniformly selected in the intergenic regions, and 33,761 of the loci were uniformly selected from the 69,022 loci over the whole genome (of which 16,457 were in genes and 17,304 were in intergenic regions). When comparing different traits, different models, and different marker sampling strategies, a fivefold cross-validation method was used to evaluate the prediction accuracy of the genomic selection model. To reduce the sampling error, each sampling method was repeated 100 times, and the “pairwise.t.test” function in *R* was used to analyze the significance of the differences.

## Results

### Developing the ZDX1 array with evenly distributed SNPs

The 158,959 high-quality SNPs (Supplemental Table 1) were evenly distributed across the 20 soybean chromosomes. The number of SNP sites on each chromosome ranged from 6086 to 9315, of which 90.23% fell within 10 kb (Supplemental Table 2). In addition, the SNP number showed a highly significant positive correlation with chromosome length, with a Pearson correlation coefficient of 0.98 (*p* = 8.61E-14) (Fig. 1b). We mapped 64,435 of the candidate SNPs to 50,592 annotated genes, accounting for 90.92% of the total number of predicted genes in the soybean reference genome (Fig. 1c). In addition, another 4.29% of the large-effect SNPs could potentially affect gene function, including 5684 nonsynonymous SNPs, 119 stoploss SNPs (four of which were both nonsynonymous or stoploss), 604 stopgain SNPs, six frameshift SNPs, and 414 alternative splicing SNPs. The SNPs selected for inclusion in the ZDX1 array also included 14,685 synonymous sites, 6120 unknown sites, 14,845 sites located in intronic regions, 12,158 sites located within 1000 bp upstream or downstream of a gene, 9804 sites located in untranslated regions, and 94,524 sites located in intergenic regions (Supplemental Table 1). A/G and T/C (transitions) represented the main nucleotide variants on the array, accounting for 68.25% of the total SNPs. The site frequency spectrum (SFS) for the 2214 re-sequenced accessions showed that the sites with minor allele frequency (MAF) > 0.1 accounted for 81.3% of the total. SNPs with MAFs between 0.10–0.20, 0.20–0.30, 0.30–0.40, and 0.40–0.50 accounted for 31.48%, 19.20%, 15.79%, and 14.85%, respectively (Fig. 1d). Collectively, the array had high gene coverage and utilization.

**Table 1:** Allelic combinations at the *rhg1-a*, *Rhg4*, and *GmSNAP11* loci

Combination	*rhg1*-a/GmSNAP18 Gm18_1643660	*Rhg4*/GmSHMT08 Gm08_8361148	*GmSNAP11 *Gm11_32970174	Number of parental lines	Number of candidate parental lines	Number of progeny
Com1	GG	GG	TT	0	6	1
Com2	CC	CC	CC	76	162	557
Com3	CC	CC	TT	0	0	3
Com4	GG	CC	TT	0	0	2
Com5	CC	GG	CC	1	0	6
Com6	GG	CC	CC	0	0	1
Com7	CG	CC	CC	0	0	1
Com8	CG	GC	TC	0	1	0

In addition, the ZDX1 array retained high-priority loci, including 2402 SNPs for genes related to important traits and 627 SNPs for genes that underwent domestication or improvement (Supplemental Table 3). In addition, it also included 953 SNPs in QTL intervals, 547 GWAS-identified SNPs (https://soybase.org), and 110,811 SNPs that differed between ecological groups (Supplemental Table 4). Moreover, 3869 SNPs from the 1.5 K BeadChip (Hyten et al. 2008) and the BARCSoySNP6K array (Song et al. 2020) were also included (Supplemental Table 5). Compared with the three high-density arrays SoySNP50K, 180 K AXIOM^®^, and NJAU 355 K SoySNP, the ZDX1 array contained 134,737 characteristic sites (Fig. 1e), with a specificity rate as high as 84.8%. In addition, 14 important functional sites (causal SNPs) related to traits such as growth period, resistance to cyst nematodes, leaf shape, pod setting habit, seed coat color, seed dormancy, and phosphorus efficiency (Supplemental Table 6) were selected for the array.

As a final step in marker selection, we evaluated the accuracy of the marker using 817 well-established breeding materials (Supplemental Table 7), and we found that the detection rate for each sample was between 84.40 and 95.98%, with an average of 95.19%. Three DNA samples were randomly selected twice, and the genotype similarity between the two repetitions was > 99.9% (Supplemental Table 8). These results indicated that the high-density ZDX1 array was both reliable and accurate.

## Screening divergent and fixed sites of soybean in breeding

**Fig. 2:**
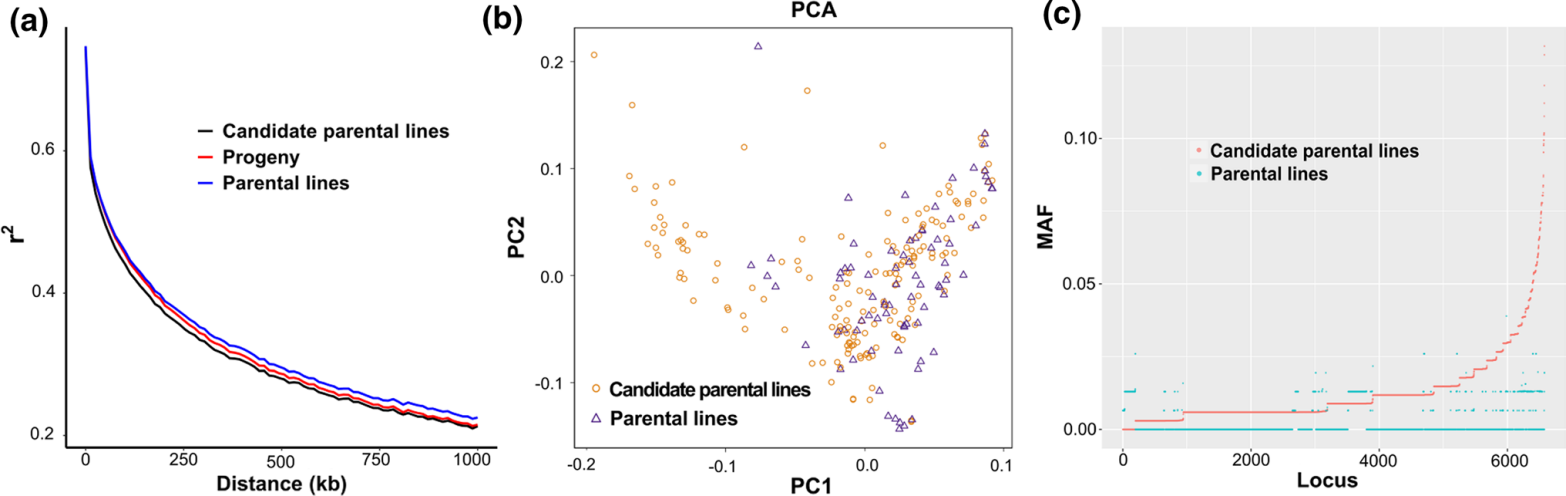
Analysis of genetic diversity of breeding population and screening of fixed sites in breeding improvement. **a** Linkage disequilibrium (LD) decay of *r*^2^ and physical distance between single nucleotide polymorphisms (SNPs) in parental lines, candidate parental lines, and progeny. **b** Principal component analysis (PCA) of 77 parental lines and 169 candidate parental lines based on kinship. Individuals from the same species are shown in the same color. **c** A scatter plot showing the minor allele frequencies (MAFs) for the parental lines and candidate parental lines at 6579 sites with the MAF of progeny = 0

Based on the phenotypic data obtained by the multi-point field identification for two years, we obtained the BLUE of 817 materials for all five traits with good phenotypic diversity. R7 ranged from 80.73 to 123.68 days, SW ranged from 12.44 to 28.16 g, protein content ranged from 37.32 to 46.50%, oil content ranged from 17.47 to 22.63%, and SY ranged from 142.53 to 533.72 g. These materials were widely distributed and phenotypically divergent. The broad-sense heritability was lowest (0.65) for plot yield among the 5 traits (Supplemental Table 9). The genetic diversity among three subpopulations was compared using LD analysis (indicated by *r*^2^), and the results showed that the attenuation rate of candidate parental line *r*^2^ values was higher than that of the progeny and parental lines, and the distances at which the *r*^2^ decayed by half were 243 kb, 279 kb, and 301 kb, respectively (Fig. 2a). These findings indicated that the candidate parental lines were helpful to broaden the genetic diversity of the parental lines. Similarly, PCA confirmed that the candidate parental lines had higher genetic diversity than the parental lines (Fig. 2b).

The percentages of fixed sites (MAF = 0) in candidate parental lines, parental lines, and progeny were 34.72%, 41.79%, and 34.63%, respectively (Supplemental Table 10, Fig. S3). In order to clarify which sites were selected and fixed during the breeding process from germplasm to elite progeny, only 6579 sites were retained, where the MAF value was 0 for progeny, 0–0.0390 for parental lines, and 0–0.1317 for candidate parental lines (Fig. 2c). A total of 235 sites were identified where the MAF value of the parental and candidate parental lines was both > 0.01, including 21 nonsynonymous SNPs and 2 stopgain SNPs in 23 important genes (Supplemental Table 11), which may have been an ideal type at the genomic level.

## Identifying elite lines with desirable traits using functional sites in the ZDX1 array

In order to select elite lines or varieties, the functionally informative SNP sites were analyzed. The *Ln/ln* locus appeared to coincide with phenotypes at a rate of almost 100%, because 649 narrow leaflet soybeans all carried the *lnln* alleles, 166 broad leaflet soybeans harbored the *LnLn* alleles, and only two soybeans segregating for broad and narrow leaflets carried *Lnln*. Interestingly, a greater proportion of round-leaf accessions was present in candidate parental lines (32.0%), while round-leaf accessions in the parental lines and progeny accounted for 10.4% and 18.4%, respectively. These proportions again reflected that breeder’s favor narrow leaflets.

We next analyzed three maturity loci of *E1*, *E3*, and *E4*, among which the *e1-fs*, *e1-as*/*e3-fs*/*e4-kes*, *e1-as*/*e3-fs*, and *e1-as*/*e4-kes* genotypes were associated with precocity (Supplemental Table 12). Notably, only one accession, Dongnong36 (80.73 d), carried the *e1-fs* genotype. Among the materials with two or more loci of *e1-as*, *e3-fs*, and *e4-kes*, nine parental lines and candidate parental lines exhibited earlier maturity (87.14–97.98 d), while three progeny (HJ15-1231, HJ15-896, and HJ15-897) had relatively late growth periods (109.17–114.32 d). These progeny may have expressed an inhibitor of early maturity.

The nematode-resistant loci of *rhg1*, *Rhg4*, and *SCN3-11* (Table 1) in the tested materials had relatively low frequencies of 1.22%, 1.71%, and 1.47%, respectively. These consisted of eight allelic combinations. A total of seven accessions carried all of the resistance loci, including three known resistant varieties, namely Kangxian1hao, Kangxian5hao, and Kangxian8hao. For the other four accessions, searching the pedigree revealed that the progenitors of HJ15-863 had resistance, while Qinong1hao, Shundou5hao, and Fengdou23 had no available information. This indicates that genotyping is the most efficient way to identify elite lines.

## Exploring optimization of parental subpopulation by integrating BI and genetic distance

**Fig. 3:**
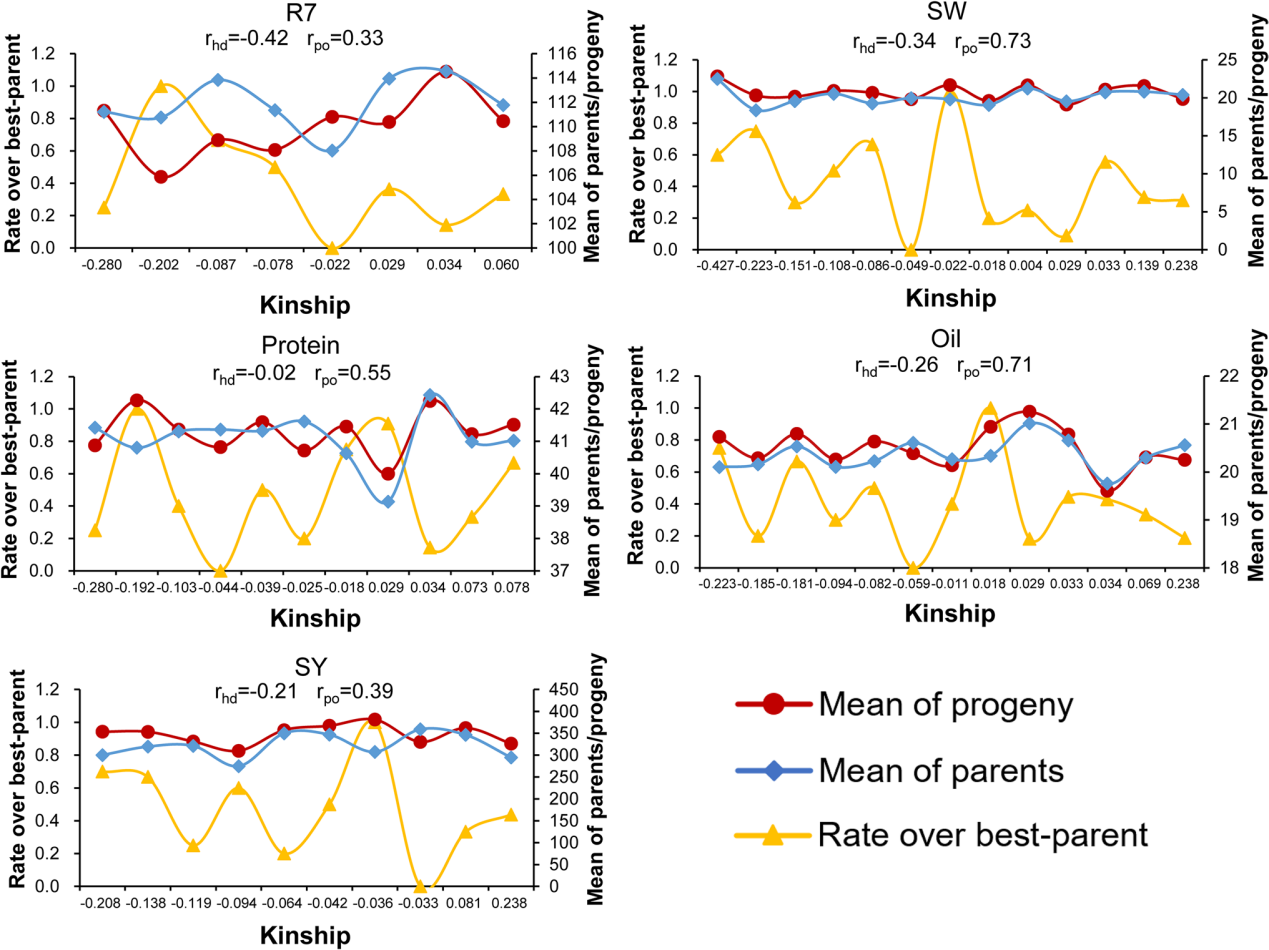
Mean value of parents and progeny, and the rate over best-parent of progeny for five traits plotted against genetic distance. The blue diamonds represent the average parental values, the red circles represent the average progeny, and the yellow triangles represents the rate over best-parent of progeny. The genetic distance is the mean value under different rate over best-parent; *r*_hd_ represents the correlation coefficient between the rate over best-parent of progeny and the genetic relationship between parents; and *r*_po_ represents the correlation coefficient between the mean value of progeny and the mean value of parents. Beginning maturity (R7), 100-seed weight (SW), seed yield (SY)

Using genotype data to generate a kinship matrix for all of the materials, pairwise genetic distances ranged between 0.54 and 2.56, with larger values indicating closer kinship (Fig. S4). Analysis of each of five traits in 298 progeny showed that the rate over best-parent was non-significantly negatively correlated with the genetic relationship between their parents (*p* = 0.30–0.97), and the correlation coefficients (*r*_*hd*_) were − 0.42 to − 0.02. This finding suggested that greater distance between parental lines resulted in a better performance potential for progeny compared to the parental lines. In addition, the mean value of each trait among progeny was positively correlated with the average parental value, with correlation coefficients (*r*_*po*_) ranging from 0.33 to 0.73, of which oil and SW appeared to be extremely significant (*p* < 0.01) (Fig. 3). These results indicate that elite progeny can be selected from hybrid combinations with elite parents.

**Fig. 4:**
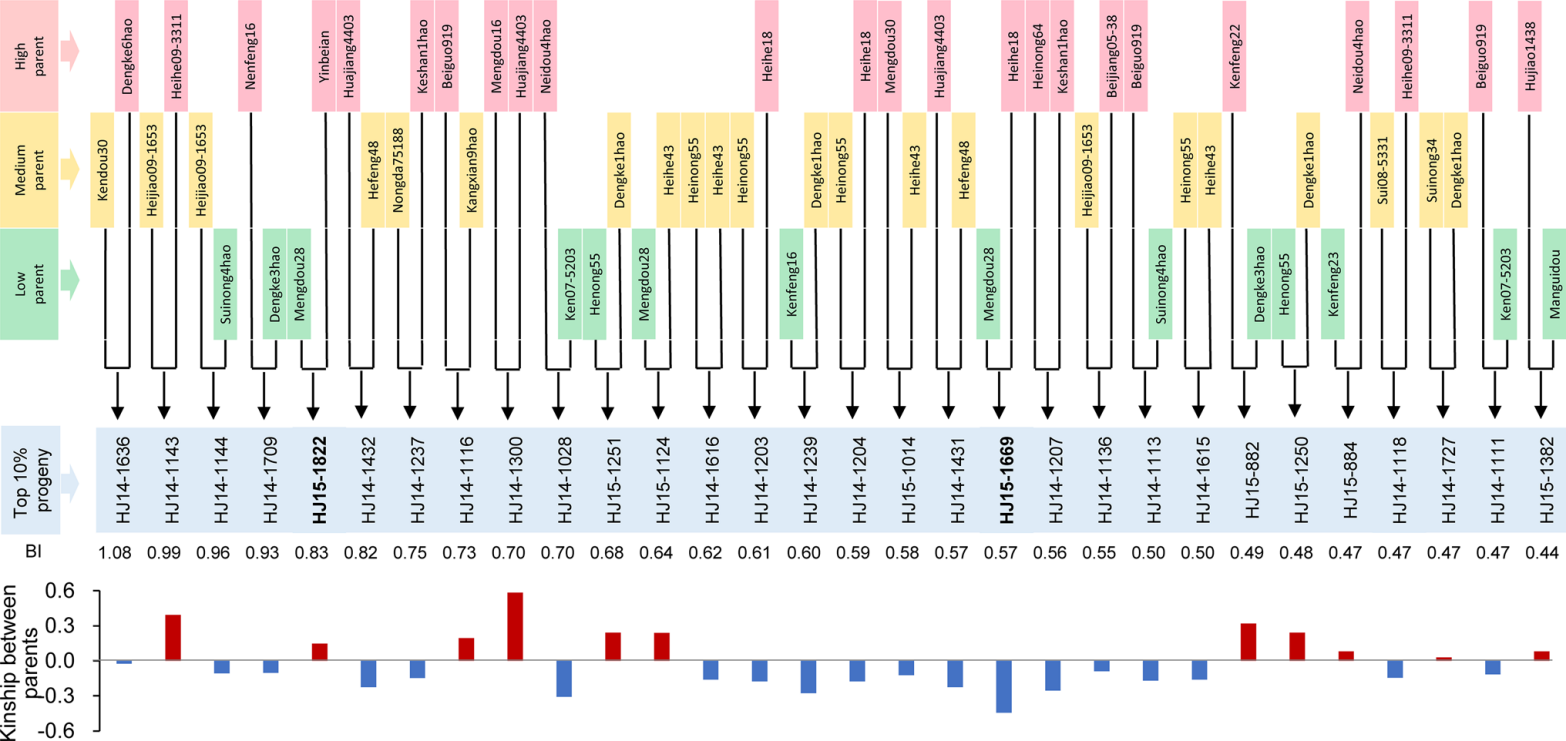
The relationship between the top 10% of progeny in multiple traits and their parental lines. The blue box in the center is the top 10% of progeny with high breeding index (BI) values. They are arranged in order from high to low from left to right. The BI values are given below the box. The parents of these lines are classified by BI value; the top third of lines with the highest BI values are the high parents; the middle third are the medium parents; and the bottom third are the low parents. The bar graph at the bottom shows the kinship between the parental lines

To select for high yield accompanied by the proper performance of the other traits, the BI was used to score the parental lines into high, medium, or low phenotypes (Supplemental Table 7). The 30 (top 10%) high-performance progeny (THP) with greater genetic distances (− 0.0298) were traced back to five types of parental combination, consisting of two progeny from high × high types, 11 progeny from high × medium types, nine progeny from high × low types, three progeny from medium × medium types, and five progeny from medium × low types. Of these THP, 73.3% were descended from at least one parent with high BI (Fig. 4). These results suggest that the selection of more distantly related parents, including at least one parent with high BI, will be more likely to produce progeny with good agronomic performance. This standard was also confirmed by developing two new varieties, Mengdou1137 and Mengdou640 which have been released for national trials.

To enable efficient breeding, the redundant parental lines were firstly eliminated. Among them, the lower 30 progeny (bottom 10%) were derived from 12 parental lines, including Dengke4hao and Hujiao1120 (Supplemental Table 13). These will not be used in future breeding. Meanwhile, compared to the parental lines (Fig. S5a), 21 candidate parental lines including Mei1 and Nenao08-1092, based on kinship scores of > 1.0, were also excluded due to small genetic distance (Supplemental Table 14). Finally, the 82 accessions with the top 10% of BI values were selected as the new parental lines to form all of the potential combinations, with distances of − 0.5 to 0 (Fig. S5b). Using a genetic distance <  − 0.3 as the standard, 46 high-potential combinations were proposed for future breeding (Supplemental Table 15). By eliminating redundant parents and designing new combinations, the development of soybean varieties will be improved in the near future.

## Improve the accuracy of genomic selection in theoretical and actual breeding based on the ZDX1 array

**Fig. 5:**
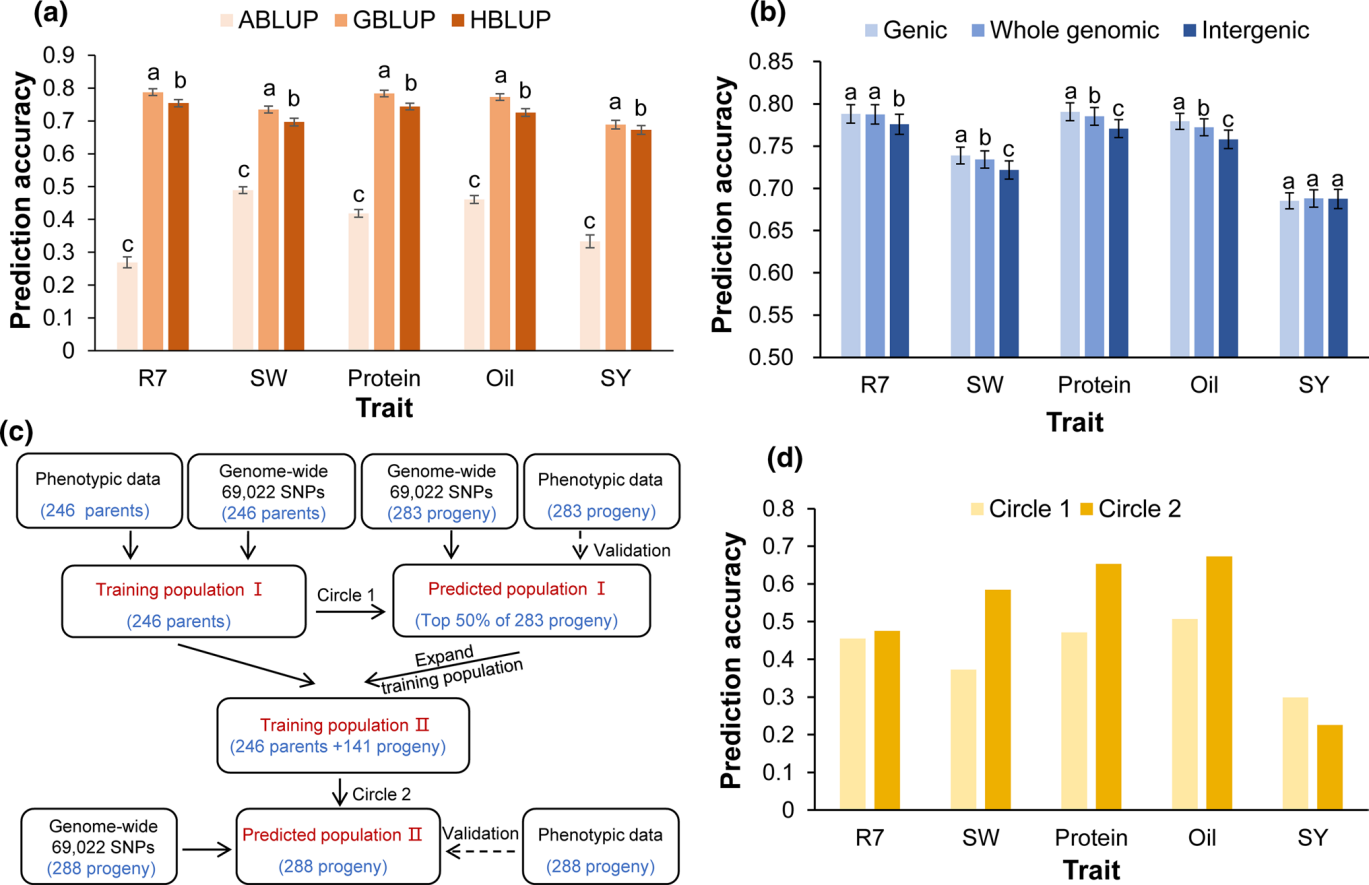
Different strategies based on the ZDX1 array in genomic selection. **a** The prediction accuracy (rGS) of three models for five traits with 100 repetitions using fivefold cross-validation. The prediction accuracy is shown as the mean value ± standard deviation. **b** Prediction accuracy of selected sites for gene region, whole genome, and intergenic region markers. The prediction accuracy is shown as the mean value ± standard deviation. **c** Simulating the process of predicting progeny performance by parental resources in actual breeding and the prediction process after using progeny to expand the training population. **d** Prediction accuracy for five traits for the 246 parents (Training Population I) and 246 parents + 141 progeny (Training Population II) used as training populations for prediction. Genomic best linear unbiased prediction (GBLUP), pedigree-based best linear unbiased prediction (ABLUP), combined best linear unbiased prediction (HBLUP), beginning maturity (R7), 100-seed weight (SW), seed yield (SY)

The results of GBLUP analysis to test the accuracy of selection based on the ZDX1 array revealed that the prediction accuracy was 0.79 for R7, 0.73 for SW, 0.78 for protein content, 0.77 for oil content, and 0.69 for SY. These scores were all significantly higher than those of ABLUP and HBLUP based on both pedigree relationship and genotype data (*p* < 0.01) (Fig. 5a, Supplemental Table 16).

We subsequently identified 33,756, 33,733, and 33,761 sites that were selected as marker subsets from gene regions, intergenic regions, or the whole genome, respectively. GBLUP analysis confirmed that these three marker sets showed no significant differences in their accuracy for predicting yield. For each of the other four traits, the accuracy of prediction using markers for genic regions was 2.33% higher than that of SNP markers for intergenic regions, with highly significant (*p* < 0.01) differences among methods. In addition, markers associated with genic regions were more accurate by an average of 0.57% compared to those sampled from across the whole genome and were significantly (*p* < 0.01) more accurate for predicting SW, protein content, and oil content (Fig. 5b, Supplemental Table 16). Furthermore, the use of only 33,756 SNPs in genic regions also significantly (*p* < 0.01) improved the predictive accuracy for selecting these three traits compared with the accuracy provided by using all 69,022 of the SNPs. In most cases, the strategy of sampling SNP markers for gene-encoding regions can reduce the number of requisite markers while improving the accuracy of genomic selection.

In order to improve the efficiency in predicting progeny of actual breeding, we first selected 246 parents as training population I and 283 of the 571 progeny bred in 2015 as predicted population I. The prediction accuracy for five traits in 141 high-value lines ranged from 0.30 to 0.45 (Circle 1). The training population I, with the 141 high-value progeny, was then expanded to generate training population II to further predict the 288 progeny bred in 2016 (predicted population II) (Fig. 5c). With the exception of yield, the predictive accuracy was improved for the other four traits, ranging from 0.48 to 0.67 (Circle 2), while the average accuracy was significantly increased by 32.1% (*p* = 0.024) (Fig. 5d, Supplemental Table 16). Collectively, the above “cycling training population” strategy greatly improved the prediction accuracy through the use of a model that established with the parental lines and continuously expanded with high-performing progeny obtained through practical breeding.

## Discussion

### Characteristics of SNPs in the ZDX1 array

The previous soybean arrays were developed based on information obtained from only a few to dozens of cultivated or wild species (Lee et al. 2015; Song et al. 2013; Wang et al. 2016). However, the use of a wide variety of accessions can reduce the possibility of losing rare alleles found only in a small number of samples. A similar strategy has been preliminarily applied in the development of arrays for other species such as *Eucalyptus* (Silva-Junior et al. 2015). The initial locus information in our ZDX1 array was derived from 2214 representative soybeans and included a core collection from soybeans originating from China. This was the biggest dataset used to date far in the development of soybean arrays. We selected SNPs not only based on high MAF (Lee et al. 2015; Wang et al. 2016), but also their positions in the genome. The uniform distribution of loci enables the SNPs in the ZDX1 to capture variation in the centromere region. In particular, the extremely high coverage of annotated genes and many important sites make the array useful for correlation analysis and genetic mapping, which has been proved in pigeon pea (Singh et al. 2020). The average distance between adjacent SNPs was 6.0 kb for ZDX1, which was much smaller than the reported extents of LD of 12 kb and 58 kb in *G. soja* and landraces, respectively (Li et al. 2019). Compared with the other three arrays, SoySNP50K, 180 K AXIOM®, and NJAU 355 K SoySNP, ZDX1 contains more than 80% unique sites. In addition, we also preferentially added functional sites related to important agronomic traits. The functional and cost-effective genotyping platform provided by the ZDX1 array will be widely used in soybean breeding and genetic research. More importantly, we made innovative use of an actual breeding population step by step and integrated high-throughput sequencing, which has provided strong guiding significance in breeding.

## Discovering novel lines and genes using functional sites in the ZDX1 array

When screening germplasm for potential use as parents, phenotypic identification is time-consuming and laborious, and the results are strongly influenced by environmental factors. Therefore, the molecular marker-assisted selection represents an efficient and effective method for screening target traits (Barabaschi et al. 2016). For the leaflet shape in the present study, the genotypes detected by functional SNPs corresponded closely to phenotypes. Parental lines had a higher frequency of narrow leaflets (89.6%), indicating that either breeders favor narrow leaflets or that local soybeans exhibit narrow leaflets in northeast China in response to environmental conditions. Another reason that this genotype is favored is that it usually has good performance, with more than four seeds per pod (Fang et al. 2013), and it enables greater light transmission through the canopy. However, the most important traits were quantitative traits controlled by multiple genes. Maturity is one of the most important traits for adaptability, and five functional sites were included in this array. Interestingly, we found that three progeny (HJ15-1231, HJ15-896, and HJ15-897) had relatively late growth periods (109.17–114.32 d) even though they carried alleles linked to early growth (*e1-as*, *e3-fs*, or *e4-kes*), indicating that they may be sources for identifying novel genes related to maturity. By identifying functional sites related to soybean cyst nematode resistance, three resistant parental lines were confirmed. In addition, we identified four novel resistant lines, among which only HJ15-863 could be traced to a resistant source progenitor, indicating that genotyping is the best method to find new soybean cyst nematode-resistant sources. In comparison with above the three traits, leaflet shape is controlled by few genes and has a higher selection rate than maturity, which is controlled by multiple genes. With an increasing number of functional markers involved in ZDX1 array, such as the soybean cyst nematode resistance gene, the array will play a much more important role in directional selection.

## Increasing the selection rate of parents and progeny for soybean improvement

Previous studies have shown that if the genetic variation and distance between accessions are sufficiently large in the parent population, then a progeny population with greater genetic variation can be obtained (Mikel et al. 2010). Although the SNP array data in this study confirmed that greater genetic distance between parents resulted in a higher rate of progeny performing better than their best-parent, we found that six elite progeny came from closely related parents with genetic distances ranging from 0.2404 to 0.5843, because there were superior local varieties with good combined abilities in their pedigree. Our results suggest a strategy for combining parents with a greater chance of obtaining excellent progeny while avoiding blindly formulating a large number of suboptimal combinations. The results of two major subpopulations in *indica* (Xie et al. 2015) showed that, in the process of domestication, the selection and fixation of target SNPs or genome regions may reflect the preference of local breeders and the adaptability of varieties to the local climate. Elite progeny have accumulated a complement of selected SNPs. By querying the Arabidopsis homologous genes corresponding to the 23 important genes mentioned above, we found that *Glyma.06G083500* was involved in regulating reproductive development, while *Glyma.06G081300* may confer drought resistance (https://soybase.org). These potentially functional genes may be used in future breeding.

In soybean genomic selection research, scientists have used genetic resources (Shu et al. 2013; Zhang et al. 2016), breeding varieties (Jarquín et al. 2014; Ma et al. 2016; Xavier et al. 2016), and germplasm and recombinant inbred lines (Matei et al. 2018; Stewart-Brown et al. 2019). Compared with some studies on the brink of operational implementation of genomic selection (Silva-Junior et al. 2015), the population used in the present study was closely related to breeding. This method is rarely reported. We innovatively simulated the cyclical process used in actual breeding practices and expanded the training group of parental lines. Using progeny with higher predicted values can greatly improve the accuracy of predictions. In this study, prediction accuracy provided by GBLUP reached an average of 0.75, which was similar or higher to that in previously reported results (Supplemental Table 17). These findings further indicate that genomic information reflected by ZDX1 can better reflect the genetic structure of the breeding population than pedigree relationships. For complex traits with low-to-moderate heritability, high-density SNPs were largely sufficient to obtain reliable predictions (Zhang et al. 2015), especially selecting a subset of highly efficient markers (e Sousa et al. 2019; Liu et al. 2019; Ma et al. 2016). However, sampling SNPs located within genic regions was more informative than sampling SNPs from intergenic or random regions in this study, suggesting that the ZDX1 array can solve the problem caused by marker effects and may be widely used for different purposes. Indeed, sampling SNPs from genic regions can ensure or even significantly improve the accuracy of prediction and reduce sequencing costs.

**Fig. 6:**
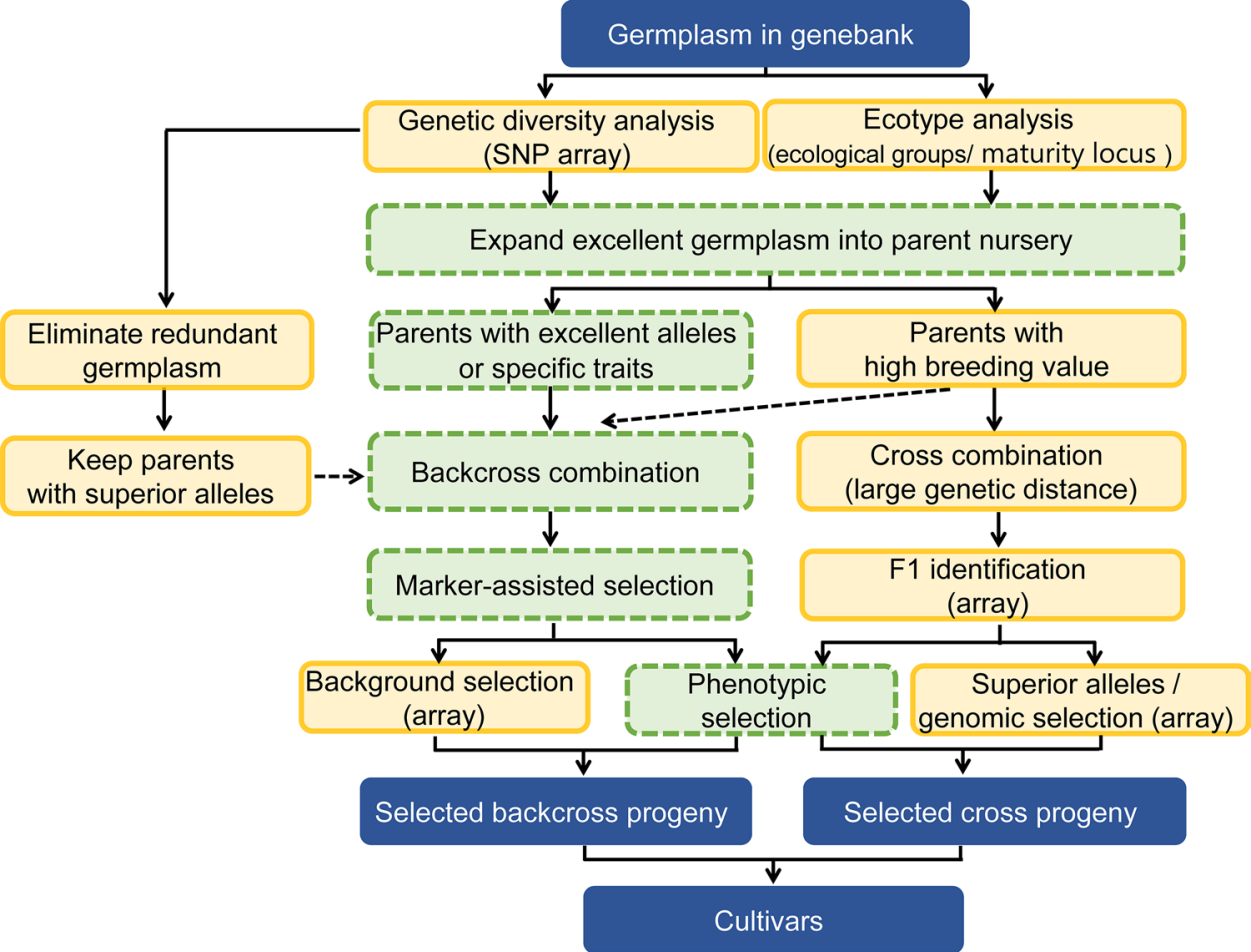
Optimized scheme for using genome-wide molecular marker breeding combined with array screening. Germplasm resources are introduced from a resource bank, redundant accessions are eliminated through genetic diversity analysis, and accessions with excellent alleles are retained. Germplasm accessions with higher breeding index (BI) values are used as one of the candidate parents in cross breeding, and the superior resources are further screened for those with highly distant genetic relationships for cross breeding. A microarray is then used for F_1_ identification, hybrid segregation combined with phenotypic selection, and whole-genome selection. Germplasm with high breeding values with multiple excellent traits can also be used as recurrent parents. When germplasm with specific traits is used for backcross improvement, functional markers can be used for foreground selection, and microarrays can be used for genome-wide background scanning, combined with phenotypes for selection, resulting in the selection of excellent stable lines. The green dashed boxes indicate the commonly used breeding method, and the boxes enclosed by solid yellow lines represent the improved scheme proposed in this study

For hybrid crops, the identification of F_1_ heterosis often involves the relationship between parent selection and progeny performance (Zhong and Jannink 2007), which is relatively rare for selfing crops such as soybean. In traditional plant breeding, breeders mainly rely on phenotype and experience, which may be confounded by a range of factors (Barabaschi et al. 2016). Molecular breeding is therefore considered the best option for improving breeding efficiency (Chen et al. 2014). However, molecular techniques have thus far failed to effectively integrate high-throughput genotyping with the whole breeding process. In this study, we propose an optimization strategy to comprehensively improve the breeding processes of parental evaluation, selection for crosses, and progeny selection using the ZDX1 array (Fig. 6). However, whenever breeders try to introduce new genetic resources, genotypes should be examined first to eliminate redundant candidate parental lines compared to the original pool of parents, and phenotypes with high BI values need to be considered in order to improve the selection efficiency for elite lines. By using the most distant parents, the distribution range of the progeny expanded and effectively increased the probability of obtaining elite progeny. Undoubtedly, the mechanism of good performance in the remaining progeny from medium × medium and medium × low crosses needs to be elucidated, which further indicates that the direct selection of parents based on phenotypes is inefficient. In this study, we combined an affordable and high-throughput functional SNP array, ZDX1, to improve conventional breeding procedures. This is a successful example of applying the principles of molecular breeding from theory to practice.

## Supplementary Information

Below is the link to the electronic supplementary material.

**Fig. S1** The types (inner circle) and geographic origins (outer circle) of the 2,214 soybean accessions used in this study. Abbreviations for the countries of origin are AUT, Austria; BEL, Belgium; BRA, Brazil; CAN, Canada; CHN, China; EE, Eastern Europe; GER, Germany; INA, Indonesia; IND, India; ITA, Italy; JPN, Japan; KOR, South Korea; NGR, Nigeria; POL, Poland; PRK, North Korea; RUS, Russia; SRB, Former Serbia and Montenegro; TAL, Thailand; UKN, Unknown; USA, United States of America; VIE, Vietnam (TIF 79131 KB)

**Fig. S2 **Typical cluster graphs of single nucleotide polymorphisms (SNPs) in ZDX1. (a–b) SNPs fall into separate clusters, representing a genotype trajectory (AA or BB). (c) The SNPs show two clearly defined clusters (AA and BB). (d) The SNPs show three clearly defined clusters (AA, AB and AB). The above mode does not need to be adjusted. There are some close clusters in the genotyping in (e1) that can be adjusted to two clusters (AA and BB) by manual correction (e2). (f1) A few loci show 4–5 different clusters, and their genotypes may be AA, AB or BB. A more accurate cluster file can be obtained by adjustment, and the two closest clusters (such as AA and AB) may be combined into one cluster (AA) (f2) (TIF 79135 KB)

**Fig. S3 **Soybean genome map. Tracks from outside to inside: the outermost circle shows the 20 soybean chromosomes along the whole genome; A, B, C, and D represent the MAF for all of the accessions, progeny, candidate parental lines, and parental lines, respectively (TIF 79135 KB)

**Fig. S4 **Kinship plot for the 817 accessions. The heatmap of the values in the kinship matrix was created using GAPIT (TIF 79133 KB)

**Fig. S5 **Screening redundant candidate parental lines and making potential combinations based on genetic distance. (a) Among 169 candidate parental lines, the number of redundant lines was determined based on the different genetic distances (0.5, 0.6, 0.7, 0.8, 0.9, 1.0) between the non-parental and parental lines. (b) Using 82 accessions that had the top 10% breeding values, the number of potential combinations was determined based on the different genetic distances (−0.5, −0.4, −0.3, −0.2, −0.1, 0) between them (TIF 79129 KB)

Supplementary file6 (XLSX 47141 KB)

## Data Availability

Genotypic data generated during the current study are available in the [SoyFGB] repository [https://sfgb.rmbreeding.cn/about/download/detail]. Phenotypic data analyzed during this study are included in this published article and its Supplementary Information files.

## Abbreviations

ABLUP: Pedigree-based best linear unbiased prediction
BLUE: Best linear unbiased estimates
BI: Breeding index
GBLUP: Genomic best linear unbiased prediction
HBLUP: Combined best linear unbiased prediction
PCA: Principal component analysis
SNP: Single nucleotide polymorphism
THP: The high-performance progeny
